# Spatial and temporal changes of parasitic chytrids of cyanobacteria

**DOI:** 10.1038/s41598-017-06273-1

**Published:** 2017-07-20

**Authors:** Mélanie Gerphagnon, Jonathan Colombet, Delphine Latour, Télesphore Sime-Ngando

**Affiliations:** 1 LMGE, Laboratoire ‘Microorganismes: Génome et Environnement’, UMR CNRS 6023, Université Clermont-Auvergne, BP 80026, 63171 Aubière Cedex, France; 2 Present Address: Leibniz-Institute of Freshwater Ecology and Inland Fisheries (IGB), Müggelseedamm 301, Berlin, 12587 Germany

## Abstract

Parasitism is certainly one of the most important driving biotic factors of cyanobacterial blooms which remains largely understudied. Among these parasites, fungi from the phylum Chytridiomycota (i.e. chytrids) are the only eukaryotic microorganisms infecting cyanobacteria. Here, we address spatiotemporal dynamics of the cyanobacterial host *Dolichospermum macrosporum* (*syn. Anabaena macrospora*) and its associated chytrid parasites, *Rhizosiphon* spp., in an eutrophic lake by studying spatial (vertical, horizontal) and temporal (annual and inter-annual) variations. Our results show homogenous chytrid infection patterns along the water column and across sampling stations. However, the prevalence of infection presented drastic changes with time, at both intra- and inter-annual scales. In 2007, a maximum of 98% of vegetative cells were infected by *R. crassum* whereas this fungal species was not reported seven years later. In opposite, *R. akinetum*, a chytrid infecting only akinetes, increased its prevalence by 42% during the same period. High chytrid infection rate on the akinetes might have sizeable consequences on host recruitment (and proliferation) success from year to year, as supported by the recorded inter-annual host dynamics (affecting also the success of other chytrid parasites). The spatial homogenous chytrid infection on this cyanobacterium, coupled to both seasonal and inter-annual changes indicates that time, rather than space, controls such highly dynamic host-parasite relationships.

## Introduction

Phytoplankton constitutes a key player in the trophic food web. Its genetic structure, diversity, and seasonal succession have been largely discussed previously^1–4^. Its seasonal succession is driven by several biotic factors such as predation^5^, competition among themselves^6, 7^ or with other food web components^8^, and parasitism^9–12^, although the later factor remains largely understudied. Among the most common pathogens of freshwater phytoplankton are fungal species that belong to the phylum Chytridiomycota (i.e. chytrids)^13^. The ability of chytrid parasites to decimate phytoplankton populations, and to promote their genetic diversification^14, 15^ make these parasites an important driving factor for the primary producers dynamic and thus, for the rest of the trophic web^7^.

During the last decade, a few studies have investigated the molecular chytrid diversity^16, 17^ and its spatial^18, 19^ or temporal^9^ distribution in freshwater lakes at the community level. However, although parasitic chytrids are totally dependent on specific hosts, to the best of our knowledge, only a single study dealt with the spatiotemporal dynamics of particular chytrid-phytoplankton pairings^20^. In this study the vertical distribution of the diatom *Asterionella formosa –*and its chytrid parasite *Zygorhizidium planktonicum*, was investigated during 1.5 years, in Lake Maarsseveen, Netherlands. Gsell, *et al*.^20^, concluded that both vertical and temporal gradients of abiotic factors could impact host-parasite interactions. However, in most freshwater lake studies on the host-parasite interactions, the central area of lake was the only monitored point^9, 21, 22^, neglecting potential horizontal spatial gradients in host-parasite dynamics. Since chytrids, such as other parasites, are known to be closely linked to their host density, the first objective of our study was to characterize an accurate host-parasite pairing distribution within the lake. Thus, we tracked the vertical distribution of the cyanobacterium *Dolichospermum macrosporum* (syn. *Anabaena macrospora*) and its chytrid parasites *Rhizosiphon crassum* and *R. akinetum*, at two contrasted stations (a deep central station (CS) and a shallow littoral station (LS) (Supplementary Fig. 1)), within the eutrophic Lake Aydat during the *D. macrosporum* cyanobacterial bloom in 2011. We analyzed both host and parasite populations by investigating host densities, the prevalence and the intensity of chytrid infection, the different stages of chytrid life cycles, as well as chytrid fecundity. The results reported in this field study showed that the focus has to be put on time instead of space for a better understanding of cyanobacteria-chytrid interactions. Consequently, the second objective of this study was to describe the inter-annual changes of the chytrid species associated to *D. macrosporum* over 7 years from 2007 to 2014, mostly based on previous works led by Rasconi *et al*.^9^ and Gerphagnon *et al*.^23^ in Lake Aydat. Changes in parasitic species observed across these 7 years suggest that beyond the commonly reported direct effect of chytrid parasitism on the decline of cyanobacterial bloom, chytrids also affect cyanobacterial bloom from year to year by infecting the resting cells.

## Results and Discussion

The cyanobacterial host community was exclusively composed of *D. macrosporum*, which largely dominated the phytoplankton community, accounting up to 87.41 ± 0.02% (i.e. mean for triplicates ± SD) of the phytoplanktonic cells on Oct. 14^th^. Based on the morphology of the sporangium and the type of infected cell (vegetative cells or akinetes), two parasitic chytrid species of *D. macrosporum* were identified*: Rhizosiphon crassum* and *R. akinetum. R. crassum* is reported mainly on vegetative cells and infects several cells with a tubular rhizoid system, whereas *R. akinetum* infected exclusively single mature akinetes without expanding to adjacent cells^23^. In addition, both species presented significant differences in terms of both prevalence of infection and abundance, with a maximal abundance of *R. crassum* approximately 11 fold lower than that recorded for *R. akinetum* in 2011. *R. crassum* infected a maximum of 0.68 ± 0.04% of vegetative cells reaching maximal sporangia density of 16 ± 8 × 10^3^ sporangia.l^−1^ recorded on Oct. 7^th^ at 2 m in the central station (CS). Because all life stages of *R. crassum* were not observed, and this chytrid presented a very low abundance in 2011, vertical and horizontal distributions of sporangia were only described with more details for the *R. akinetum*-*D. macrosporum* pairing.

### A stratified host community homogenously infected

**Figure 1:**
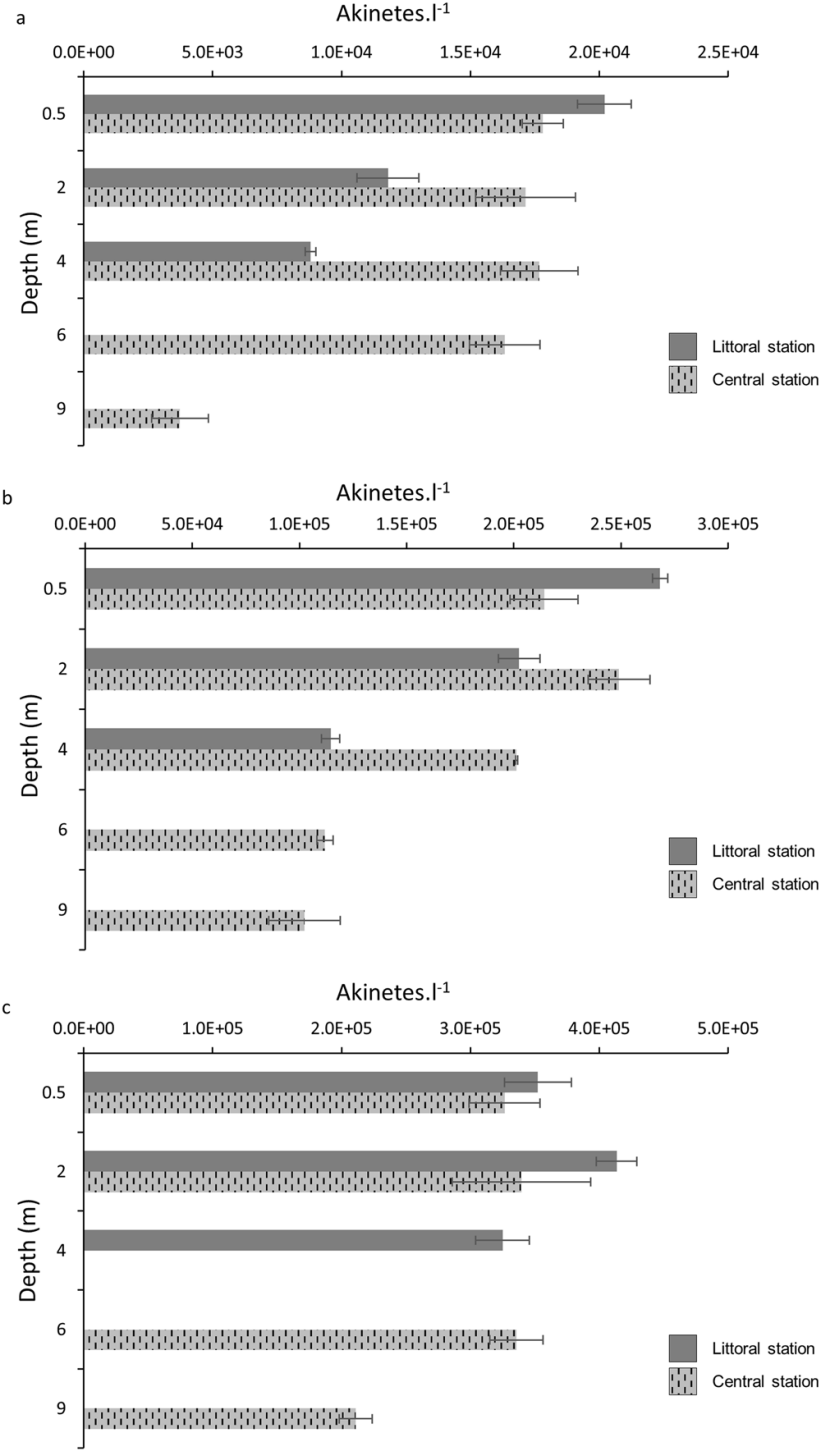
Abundances of mature akinetes. Data are reported for Central (hashed bars) and Littoral (solid bars) stations the 7^th^ (**a**), 14^th^ (**b**) and the 21^st^ (**c**) of October 2011. Error bars indicate the standard deviations.

Akinetes distribution in the water column did not show any significant difference within the two stations (CS and LS) (Mann-Whitney; *P* > 0.05). In the same way, we did not report significant differences in vegetative cell abundances. Nonetheless, we noticed an interesting difference by taking into account the vertical distribution since akinete abundances were significantly lower at the deepest layers regardless of the stations (Mann-Whitney; P = 0.03, Fig. 1). For example, on Oct. 14^th^, 2.65 ± 0.21 × 10^5^ ak.l^−1^ and 2.68 ± 0.36 × 10^5^ ak.l^−1^ were recorded at 0.5 m at CS and LS, respectively, whereas less than the half were reported for the deepest depths (CS: 1.12 ± 0.22 × 10^5^ ak.l^−1^ and 1.02 ± 0.03 × 10^5^ ak.l^−1^ at 6 m and 9 m, respectively, and LS: 1.15 ± 0.03 × 10^5^ ak.l^−1^ at 4 m). This trend was reported for all dates and stations but was less pronounced at the last sampling date, probably due to an increase of the mixing layer (from 5 m on Oct. 7^th^ to 8 m on Oct. 21^st^).

**Figure 2:**
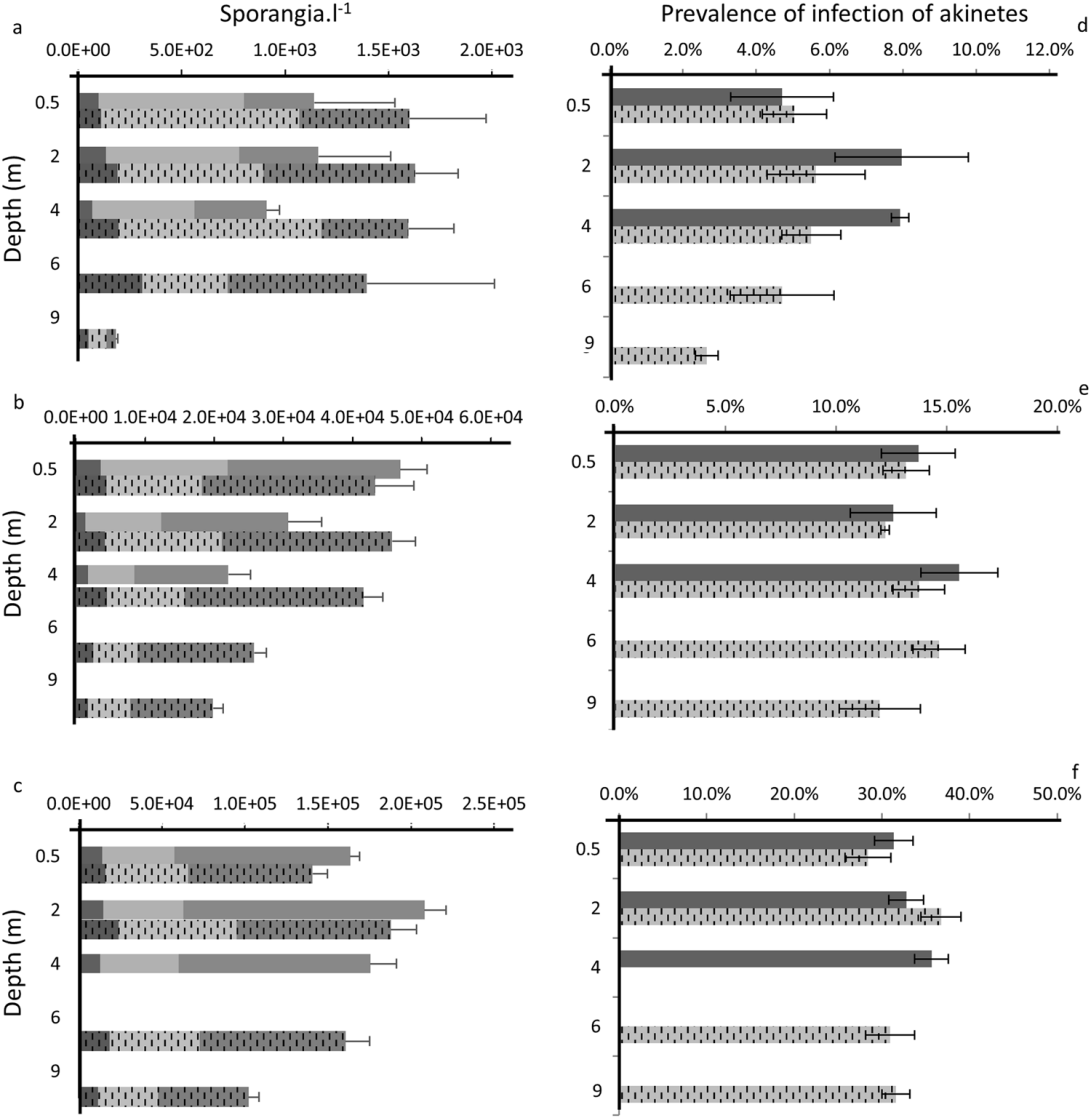
Distribution of *R. akinetum*. Vertical and horizontal distributions of the life phase abundance of *R. akinetum* (left column: Young phase (grey), Mature phase (light grey), Empty phase (dark grey)) and Prevalence of infection of akinetes (right column) on the 7^th^ (**a**,**d**), 14^th^ (**b**,**e**) and the 21^st^ of October 2011 (**c**,**f**) at Central (hatched bars) and Littoral (solid bars) stations. Error bars represent the standard deviation for three replicates.

Similar to other parasites, chytrids are known to be closely linked to their host density^24–26^. Thus, we attempted to elucidate if akinetes and their parasites *R. akinetum* were produced simultaneously in the same place. Based on akinetes/vegetative cells ratios (Supplementary Fig. 2) it appears that akinetes differentiation and maturation first occur in the upper layers of lake. Despite numerous studies on this topic, the external factor triggering the process of akinete differentiation is still not well understood. Nutrient depletion^27–29^, low temperature^30, 31^ and high light intensity^32^ have been identified as drivers inducing akinete differentiation. In our study, the temperature was homogenous along the epilimnion, allowing its mixing. Thus, we may hypothesize that the nutrient concentrations would not have been enough contrasted to explain such akinete distribution. Light availability is higher at the lake surface which may explain why akinetes were produced in the upper layer of the water column. Light intensity is known to play a crucial role in the chytrid infection as well^24^. Also, fungi exhibit phototaxis^33^, implying that infection parameters (Prevalence (Pr) and Intensity (I) of infection) should be higher in the upper water layers. However, for all the sampling periods, except for one-time point and depth (on Oct. 7^th^ at 9 m), a homogenous fungal epidemic along the water column was reported (Fig. 2) suggesting that phototaxis would be a minor driving factor for the vertical distribution of *R. akinetum* infection. Van den Wyngaert *et al*.^34^ investigated the role of chemotaxis in chytrid-phytoplankton interactions. These authors showed that chemotaxis becomes more relevant under low host density condition due to decreasing chance contact rates between host and parasite. Then, the uniform fungal infection observed along the water column may be explained by the high capacity of *R. akinetum* to detect its host even at low host densities, as reported in the deepest water layers.

### *R. akinetum* infection efficiency: when time prevails on space

The chytrid infection was spatially homogenous during the sampling period in 2011, but presented significant temporal variations. Indeed, only 4.7 ± 1.2% of akinetes were infected by chytrids at the first date whereas an average value of 31.9 ± 3.2% was reported along the water column at CS, on Oct. 21^st^ (Fig. 2a–f).

Moreover, the intensity of infection did not reveal any difference between the first two dates but presented significantly higher values within the lake at last one (1.42 ± 0.06 spor.ak^−1^; P = 0.04). Hence, the *R. akinetum* population significantly increased throughout the sampling period (P = 0.018) from an average along the water column of 1.12 ± 0.23 × 10^3^ spor.l^−1^ on Oct. 7^th^ to 1.33 ± 0.08 × 10^5^ spor.l^−1^ on Oct 21^st^. Additionally, all *R. akinetum* parasitic life cycle stages were observed for each date in all samples and the comparison of their relative abundances did not reveal significant vertical or horizontal differences (Fig. 2a–c), highlighting a synchronized parasite population within the two sampled stations.

**Figure 3:**
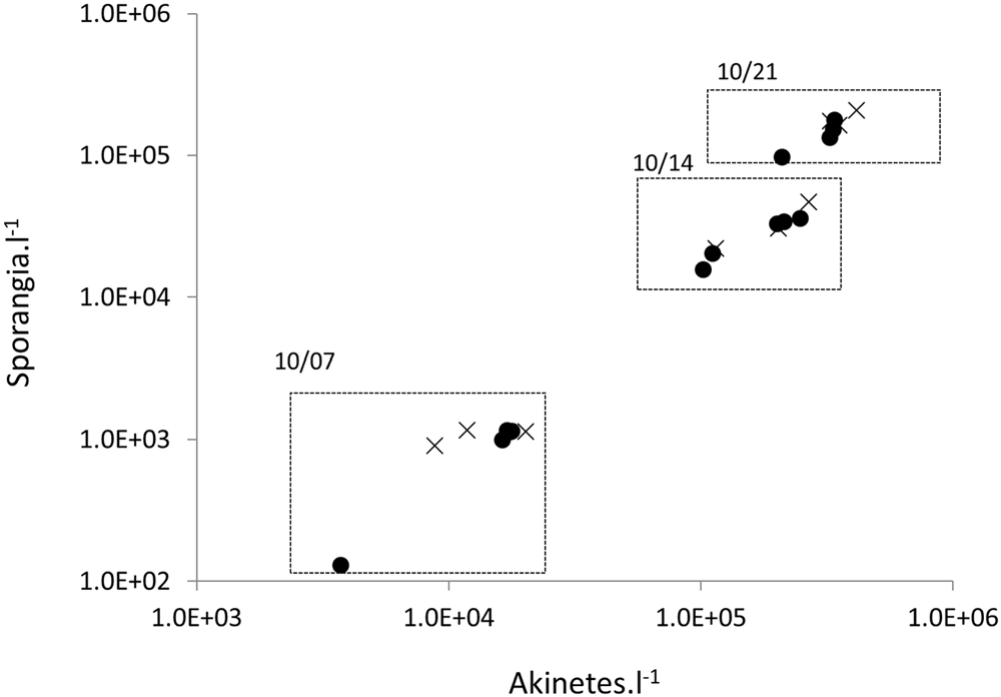
Host-parasite abundances. Relationships established between sporangia and akinete abundances at Central (circles) and Littoral (cross) stations on the 7^th^, 14^th^ and 21^st^ of October 2011.

The chytrid population increase was not linear and presented a significantly higher increase during the first seven days. The density of chytrids increased approximately 30-fold between the 7^th^ and the 14^th^ of October, but only 5-fold during last week (Figs 2a,b and 3). The first important increase of *R. akinetum* could be explained through the combination of both the life cycle duration and the success of infection. Based on the life cycle duration that we previously estimated at 3 days for *R. crassum* in the same lake^23^, we can expect the achievement of at least a whole life cycle in one week for *R. akinetum*. On 7^th^ October, *R. akinetum* presented a majority of sporangia involved in the maturation phase (48.6 ± 9.4% and 57.2 ± 3.3% for CS an LS, respectively (Fig. 2b). At this date, the biovolume of mature sporangia averaged 1749.8 ± 618.1 µm^3^ at CS and did not differ (P > 0.05; Kruskal-Wallis) from the value observed at the LS (1647.7 ± 594.9 µm^3^). By using the conversion factor^35^ (CF) of 0.0172 zoosp.µm^3^ we established a theoretical capacity of each infecting sporangium to produce 30.01 ± 10.61 and 28.3 ± 10.2 zoospores for CS and LS, respectively. Bruning^24^ reported a mean infective lifetime of *Rhizophydium planktonicum* zoospores roughly about 8 days under laboratory conditions. Once released, these zoospores infect akinetes, explaining the dominance of young stages among the population of *R. akinetum* one week later on Oct. 14^th^ (Fig. 2b, 59.9 ± 4% and 58.1 ± 4.1% for Cs and LS, respectively). The 30-fold increase in the sporangia abundance observed during the first week (which is in the same range than the estimated zoospore content) combined with the composition change of *R. akinetum* population, suggests that almost all zoospores released in their environment may have caused successful infections within a week, illustrating efficient transmission of chytrid infection for this period. Chytrid fecundity did not significantly differ between the October 7^th^ (30 ± 9 zoosp.spor^−1^) and the October 14^th^ (26 ± 6 zoosp.spor^−1^). Then we could expect an increase of the *R. akinetum* population roughly about 30-fold, as reported between the first dates. However, the chytrid population reported on the last date was only 5-fold higher that of Oct. 14^th^. Then, it appeared that only one over the six zoospores putatively released on Oct. 14^th^ would have been responsible for a successful infection on Oct. 21^st^.

The infection efficiency is submitted to diverse factors including the susceptibility of the host, its density, the temperature, the light, the nutrient concentration, as well as the grazing pressure^25, 36–38^. Among these different factors, the temperature is the unique abiotic factor driving the chytrid parasitism at each life phase. It impacts the sporangia maturation time, the zoospore infective lifetime and potentially the number of zoospores per sporangium^25^. Between the first two dates of our sampling, temperatures in the epilimnion did not vary significantly (−1 °C), whereas the last week was characterized by a sudden temperature drop (−3 °C). Such temperature decrease could impact the infective life time of the zoospores of *R. akinetum*, reducing transmission efficiency. Bruning^25^ showed that the infective life time of the zoospores of *Rhizophydium planktonicum*, a parasite of the diatom *Asterionella formosa*, was reduced by a temperature increase of 4 °C. Also, Ibelings *et al*.^21^ showed that mild winters have strong influences on both host (*A. formosa*) and parasite (*Zygorhizidium planktonicum*) which consequently does not reach epidemic levels. Diatoms are known to grow better at lower temperatures than cyanobacteria and green algae^39, 40^. We thus hypothesize that life cycle traits (i.e. temperature optima) of parasitic chytrids vary according to their hosts. This suggestion is supported by a previous study on *Rhizophydium sphaerocarpum*-*Spirogyra sp*. pairing, where authors reported that optimal conditions for chytrid infection of *Spirogyra sp*. was 30 °C^41^ which was largely higher than what was described for other algal parasites like *R. planktonicum* ^25^. A loss of zoospores, resulting in a chytrid infection reduction, can also be due to zooplankton grazing. In Lake Aydat, zooplankton community associated to cyanobacterial bloom is mainly composed of cladocerans (*Daphnia sp*., and *Ceriodaphnia sp*., Thouvenot A., personnal. communication). Kagami *et al*.^42^, Agha *et al*.^43^, and Schmeller *et al*.^44^ showed that both cladocerans^43, 45^ and protozoa^44^ can actively graze and/or grow on zoospores. As our last sampling week was marked by the decline in the cyanobacterial bloom, corresponding usually to an increase of zooplankton community^46^, we cannot reject the hypothesis of a massive grazing loss of zoospores between the 14^th^ and the 21^st^ of October.

### An overview of *D. macrosporum*-*Rhizosiphon* spp. pairings changes over 7 years

**Figure 4:**
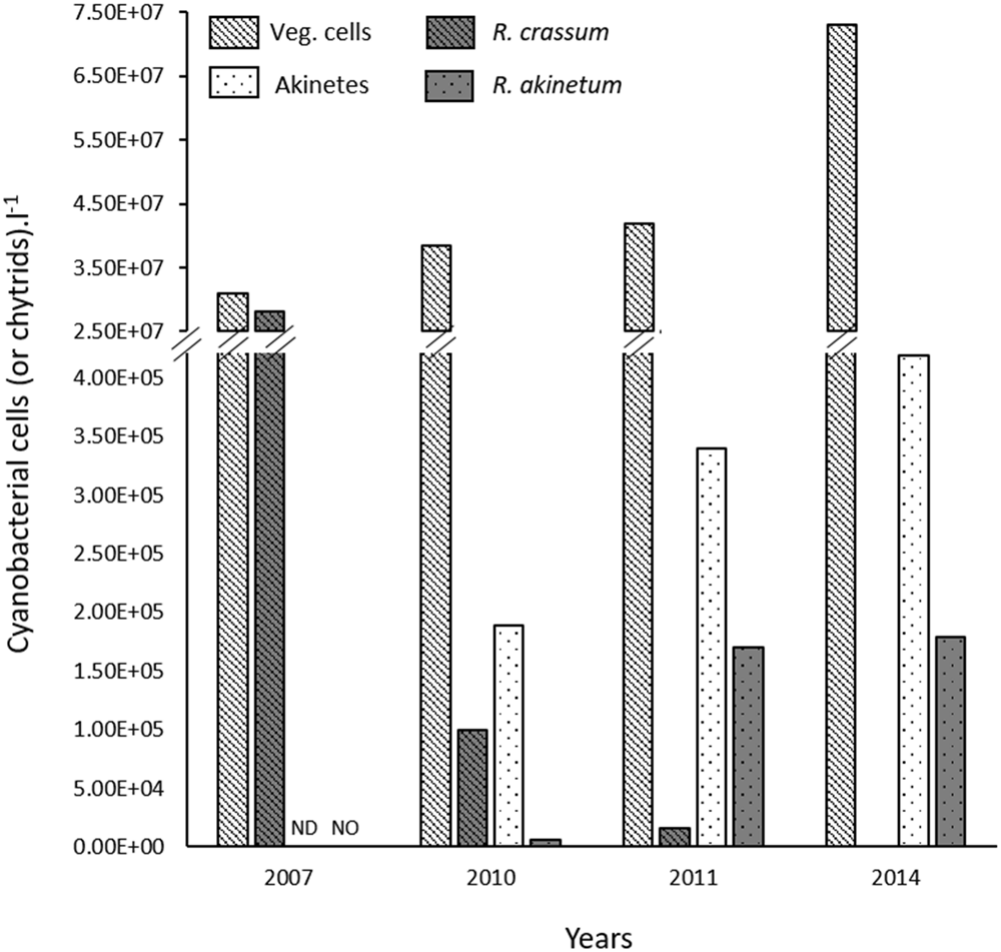
Overview of *D. macrosporum* abundances and its associated chytrids over 7 years. Maximum abundances of *Dolichospermum macrosporum* vegetative cells (veg. cells; hatched white bars) and akinetes (dotted white bars); and of their respective associated parasites, *Rhizosiphon crassum* (hatched grey bars) and *R. akinetum* (dotted grey bars), recorded in 2007, 2010, 2011 and 2014 in Lake Aydat at the Central station. ND: Not Determined; NO: Not observed.

At the annual scale, chytrid infection is homogenously distributed within the lake, independently of their location in the water column. Based on this result, and with the aim to get an idea on the *Rhizosiphon sp*. - *D. macrosporum* changes in Lake Aydat, we analyzed data on these pairings for a 7-year period from 2007 to 2014. Globally, it appears that the infection of *D. macrosporum* akinetes by *R. akinetum* highly increased whereas parasitism of vegetative cells by *R. crassum* dropped (Fig. 4).

In 2007, Rasconi *et al*.^9^ reported a maximum of 2.8 × 10^7^ *R*. *crassum* spor.l^−1^ infecting up to 98% of cyanobacterial vegetative cells (veg. cells) while *R. akinetum* was not observed. Three years later, similar maximum *D. macrosporum* cells were reported (Fig. 4). Nonetheless, maximal density of *R. crassum* deacreased (1.01 × 10^5^ spor.l^−1^) and only 6% of veg. cells were infected (Fig. 4). In contrast, *R. akinetum* was reported for the first time and presented a density of 6.3 × 10^3^ spor.l^−1^ infecting a maximum of 4.3% ± 0.5% of akinetes^23^. One year later, (i.e this study) *R. akinetum* was observed on a maximum of 36.7 ± 2.2% of akinetes while *R. crassum* showed a population density approximately 11-fold lower with a maximal prevalence of infection of 0.68 ± 0.04%. In 2014, this trend was confirmed as *R. akinetum* infected up to 42% of akinetes (i.e. 1.8 × 10^5^ spor.l^−1^) while *R. crassum* was not observed despite the intensity of *D. macrosporum* bloom was doubled (3.1 × 10^7^ cells.l^−1^ in 2007 and 7.3 × 10^7^ cells.l^−1^ in 2014) (Fig. 4).

Akinetes are the only cells which overcome unfavorable conditions and lead to the colonization of the water column by Nostocalean cyanobacteria when favorable conditions for growth return^47^. Legrand *et al*.^48^ investigated the proportion on live (intact) and dead (lysed) akinetes of *D. macrosporum* in surface sediment after the sedimentation of the entire cyanobacterial bloom in Lake Aydat (December 2014). Then, we investigated the chytrid parasitism on akinetes from their sediment samples from the Central Station (for more details about the sampling strategy see Legrand *et al*.)^48^. On the 92.3% of lysed akinetes reported we showed that 45.6% were due to *R. akinetum* parasitism. By impacting akinetes, the fungal parasitism could be responsible for an important loss of the inoculum size, and thus could delay or be responsible for a decrease in the competitive ability of their next year’s cyanobacterium host populations. Actually, Kravchuk *et al*.^49^ underlined that the size of the inoculum (akinete density in sediment) is a critical factor determining the dominance of *Dolichospermum flos aquae* in the phytoplankton community. Additionnally, Tsujimura *et al*.^50^ suggested that the start of bloom formation was related to the quantity of cyanobacteria colonies in the sediment. In Lake Aydat, increasing infection by *R. akinetum* results in increasingly severe akinete losses, which likely reduces the pool of overwintering inoculum, and might delay bloom formation. The long term survey of the phytoplankton community in Lake Aydat is consistent with this idea, showing a shift of the blooming period (threshold fixed at 40 µg eq. chlo *a*.l^−1^) from the end of August in 2007^9^ to the end of October 7 years later (Sept. 1^st^ 2009, Oct. 9^th^ 2010, Oct. 14^th^ 2011, and Oct. 20^th^ 2014). The delay of the cyanobacterial bloom establishment obsverved may have also been due to different temperatures, or mixing regime. The comparison of the mean temperatures recorded in the epilimnion before each annual bloom do not present high difference with the exception of 2009. In August 2009, the mean temperature recorded in the epilimnion was 20.3 °C whereas the ones reported in September 2010, 2011 and 2014 were 15.4 °C, 15.6 °C, and 14.9 °C, respectively. Nonetheless, the cyanobacterial population reported from 2007 to 2014 showed a rise of its maximal density (Fig. 4), underlining that both abiotic and biotic conditions were optimal for bloom development. However, the bloom duration decreased from one month in 2007 to one week only in 2014, narrowing the window of opportunity for infection of vegetative cells and could be one explanation for the *R. crassum* decrease. In 2015, one year after the maximal chytrid infection reported on akinetes, the cyanobacterial population did not exceed 10 µg eq. chl *a* l^−1^. Although this change can be partly explained by the inter-annual variations of abiotic factors, the parasitism on more than a third of resting cells reported in 2014 has to be considered as an important driver of the “non-bloom” situation observed one year later.

To conclude, it clearly appears that time prevails on space in such highly dynamic relationships. This finding suggests that sampling strategies aiming to capture the dynamics of cyanobacteria-chytrid pairings should focus on temporal, rather than spatial resolution. Nonetheless, we recommend that studies on sediment compartment should be maintained to clarify the importance of benthic phase in the entire chytrid life cycle. Inter-annual variations in chytrid infection highlight the importance of long term monitoring of chytrid-phytoplankton pairings to obtain a global view of the system. Behind the influence of the host density, different interwoven factors and processes underpin the inter-annual variations. Here we show that such variations partly result from the impact of parasitism on resting cells pool, which modulate not only host densities, bloom intensity, but could also impact the success of other chytrid parasite infecting the same host species.

## Materials and Methods

### Study site and sample collection

Samples were collected in Lake Aydat (45°39′48″N, 002°59′04″E), a small eutrophic lake (Z_max_ = 15 m, surface area = 60 ha) with a large catchment area (3 × 10^4^ ha) located in the French Massif Central region, where recurrent blooms of cyanobacteria occur in late summer and early autumn. Based on earlier works^23, 51, 52^, we sampled the cyanobacteria bloom weekly at three contrasting dates: (i) during the increasing phase of the cyanobacterial bloom known as the phase when chytrid infection starts (October 7, 2011), (ii) when the cyanobacterial bloom peaks (October 14, 2011), coinciding with the increasing fungal infection, and (iii) during the decline of the cyanobacterial bloom (October 21, 2011) when the chytrid infection reaches its maximum. For each date, two stations were sampled: the central (CS) and the littoral (LS) station of the lake (Fig. 1). The central point corresponds to the area of maximum depth, whereas the maximal depth of the littoral sampling station was 5 m. For each date, the CS was sampled at five different depths (0.5, 2, 4, 6 and 9 m), and LS at three different depths (0.5, 2 and 4 m). For each sampling depth, 20 liters of lake water were sampled using an 8-L Van Dorn bottle. To eliminate the metazoan zooplankton, immediately after being collected the samples were prefiltered through a 150 µm-pore-size nylon filter, poured into clean transparent recipients, and then transferred to the laboratory for processing. The ≥150-µm fraction was checked to make sure that it did not contain any cyanobacterium. Back in the laboratory, samples were treated: (i) to study the host community (triplicate180-ml aliquots of the raw samples were fixed with Lugol’s iodine (Sigma catalog no. 62650)), (ii) to investigate both the prevalence and the intensity of infection as well as the chytrid fecundity using a double staining method^35^.

### Physical parameters

For each sampled depth and station, water transparency was measured *in situ* using a Secchi-disk (Z_s_) and the depth of the euphotic zone (Z_eu_) was calculated according to Reynolds^53^: Z_eu_ = 1.7 × Z_s_. Temperature and dissolved oxygen profiles were obtained using a multiparametric probe ProOdO^TM^ (Ysi, Germany). A vertical pigment profile was obtained by using a BBE Fluoroprobe® (Moldaenke, Germany) (Supplementary Fig. 3).

### Host community analysis

Triplicate 180-ml aliquots of raw samples were fixed with Lugol’s iodine. For each replicate, 5 to 20 ml (depending on the phytoplankton density) were allowed to settle overnight in a counting chamber. The cells were then counted under an epifluorescence microscope (Zeiss Axiovert 200 M) following the classical Utermöhl method^54^. The entire counting chamber was inspected and *D. macrosporum* filaments, vegetative cells and mature akinetes were quantitatively analyzed. The distinction between mature and immature akinetes was based on their morphology (the presence of an outer envelope layer is characteristic of mature akinetes), shape (mature akinetes are ovoid whereas immature akinetes are spherical)^29^, and size (16-23 µm width and 21-28 µm length for the ovoid mature akinetes *vs* 13-17 µm diameter for spherical immature akinetes).

### Chytrid parasitism

For chytrid infection parameters, samples were treated following the size-fractionated community method developed by Rasconi *et al*.^55^. Briefly, 18 L of sampled water was concentrated on 25 µm pore size nylon filter. Large phytoplankton cells (≥25 µm), including the filamentous cyanobacteria *D. macrosporum*, were collected by washing the filter with 0.2 µm-pore-size-filtered lake water, fixed with formaldehyde (2% final concentration), and an aliquot of 195 µl was stained for the chitin wall. The chitin walls stained with CFW were examined using UV excitation (405 nm). We carried out the observations under an inverted epifluorescence microscope Zeiss Axiovert 200 M at ×400 magnification.

We systematically inspected 200 filaments, comprising 2480 to 4996 individual cells of *D. macrosporum* to determine the number of infected and non-infected vegetative cells and filaments. In addition, we inspected 300 mature akinetes for the number of infected and non-infected akinetes. Each sample was analyzed in the original triplicates collected. Infection parameters were calculated according to the formula proposed by Bush *et al*.^56^. These parameters include the prevalence of infection (Pr), i.e., the proportion of individuals in a given population with one or more fixed sporangia or rhizoids, expressed as Pr (%) = [(*N*_i_/*N*) × 100], where *N*_i_ is the number of infected host cells (or filaments or akinetes), and *N* is the total number of host cells (or filaments or akinetes). The second parameter is the mean intensity of infection (I) calculated as I = N_p_/N_i_, where N_p_ is the number of parasites, and N_i_ the number of the infected individuals within a host population.

Moreover, for each chytrid encountered, its life stage (stage 1 to 6) was noted and assigned to Young, Mature or Empty phase, as described in Gerphagnon *et al*.^23^. For each mature and empty sporangium, the biovolume of the sporangia was calculated by assimilating sporangia to spheres^57^. From the biovolume of mature and empty sporangia, we calculated the theoretical zoosporic content by using the Conversion Factor (CF) of 0.0172 zoospores per µm^3^ of sporangium of *Rhizosiphon akinetum* established in a previous study^35^.

To get an overview on the inter-annual changes of the chytrid parasitism associated to cyanoacterial blooms in Lake Aydat, we compared the results obtained in 2011 (i.e. this study) with the reports made in 2007^9^ and 2010^23^. Also, we used the samples collected for the long term survey of the phytoplankton community in Lake Aydat in 2014 and 2015. Basically, this survey consists to a bi-weekly sampling of the euphotic water column at the central station of the lake with a plankton net (25-mm mesh size) and a vertical pigment profile is obtained by using a BBE Fluoroprobe® (Moldaenke, Germany). Samples were kept in lugol and used to investigate chytrid parasitism and its *D. macrosporum* host population as described above.

To investigate the prevalence of infection of akinetes in the surface sediment we followed the akinete extraction method developed by Legrand *et al*.^48^. Three hundred mature akinetes were inspected and Pr was investigated by staining the chitin walls of *R. akinetum* with CFW (4% vol/vol). Samples were examined using UV excitation (405 nm). We carried out the observations under an inverted epifluorescence microscope Zeiss Axiovert 200 M at ×400 magnification.

### Statistical analyses

Because of non-normal data, the non-parametric Kruskal-Wallis test was used to test the spatial (vertical and horizontal) variations of each variable followed by a Mann-Whitney pairwise comparison with the Bonferroni correction. All statistical analyses were conducted using PAST.

## Electronic supplementary material

Supplementary Material
