# Supplementary material for: Spatial and temporal changes of parasitic chytrids of cyanobacteria
Source: Sci Rep. 2017 Jul 20;7:6056. doi: 10.1038/s41598-017-06273-1 (PMC5519717; doi:10.1038/s41598-017-06273-1)
Supplement: Supplementary file 1 — Supplementary Material [file 41598_2017_6273_MOESM1_ESM.pdf]

Supplementary Material

Spatial and temporal changes of parasitic chytrids of cyanobacteria

Gerphagnon Mélanie [1,†*], Colombet Jonathan[1], Latour Delphine[1], Sime-Ngando Télesphore[1]

[1]LMGE, Laboratoire 'Microorganismes: Génome et Environnement', UMR CNRS 6023, Université Clermont-Auvergne, BP 80026, 63171 Aubière Cedex, France

[†] *Present address: Leibniz-Institute of Freshwater Ecology and Inland Fisheries (IGB), Berlin, Germany*

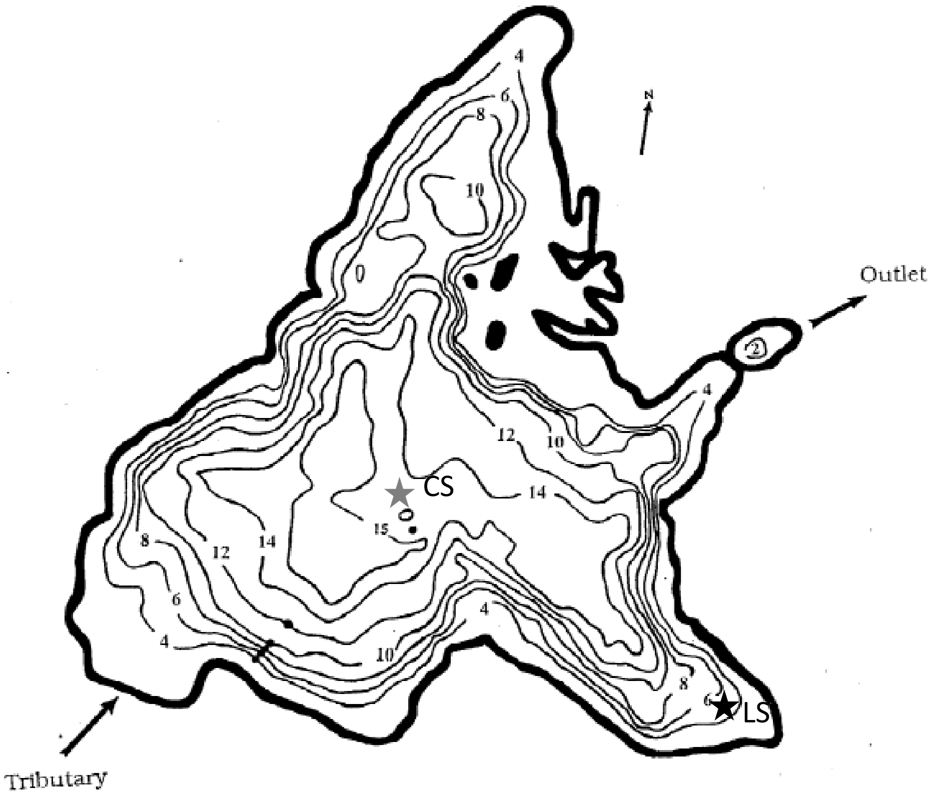

Supplementary Figure 1: The bathymetric map of Lake Aydat (reproduced with permission from Rabette and Lair, 1998) display the location of the two sampling stations: Central (CS; grey star) and Littoral Station (LS; black star). On the bathymetric map, the arrows show the flux of water and the numbers indicate the depth in meter.

Rabette, C. & Lair, N. Spatial and temporal distribution of benthic stages of *Cyclops vicinus* and *Chaoborus flavicans* in relation to abiotic factors and benthic fauna. Hydrobiologia, **390**, 61-72. (1998).

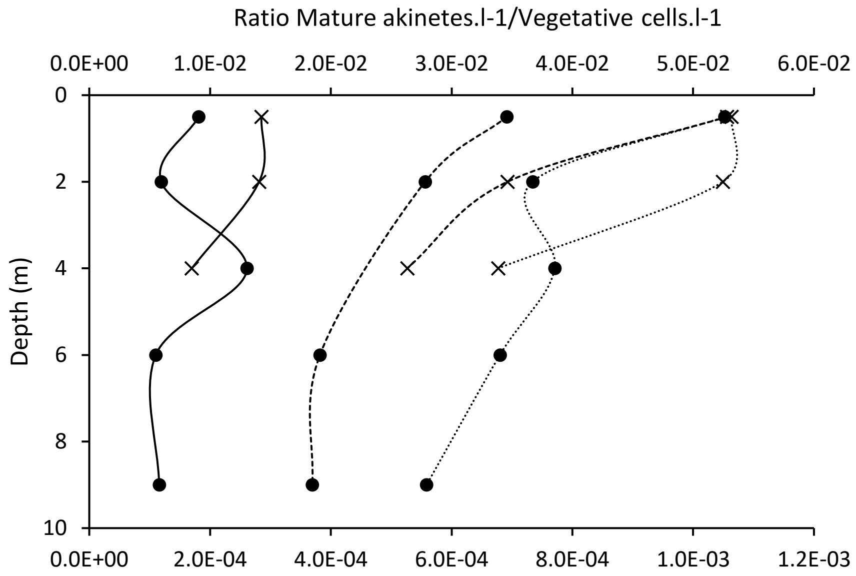

Supplementary Figure 2: Ratio of mature akinetes/vegetative cells of *Dolichospermum macrosporum* reported at Central (circles) and Littoral (cross) stations in lake Aydat on the 7th (dotted lines) 14th (solide lines) and 21st (dash lines) of October 2011.

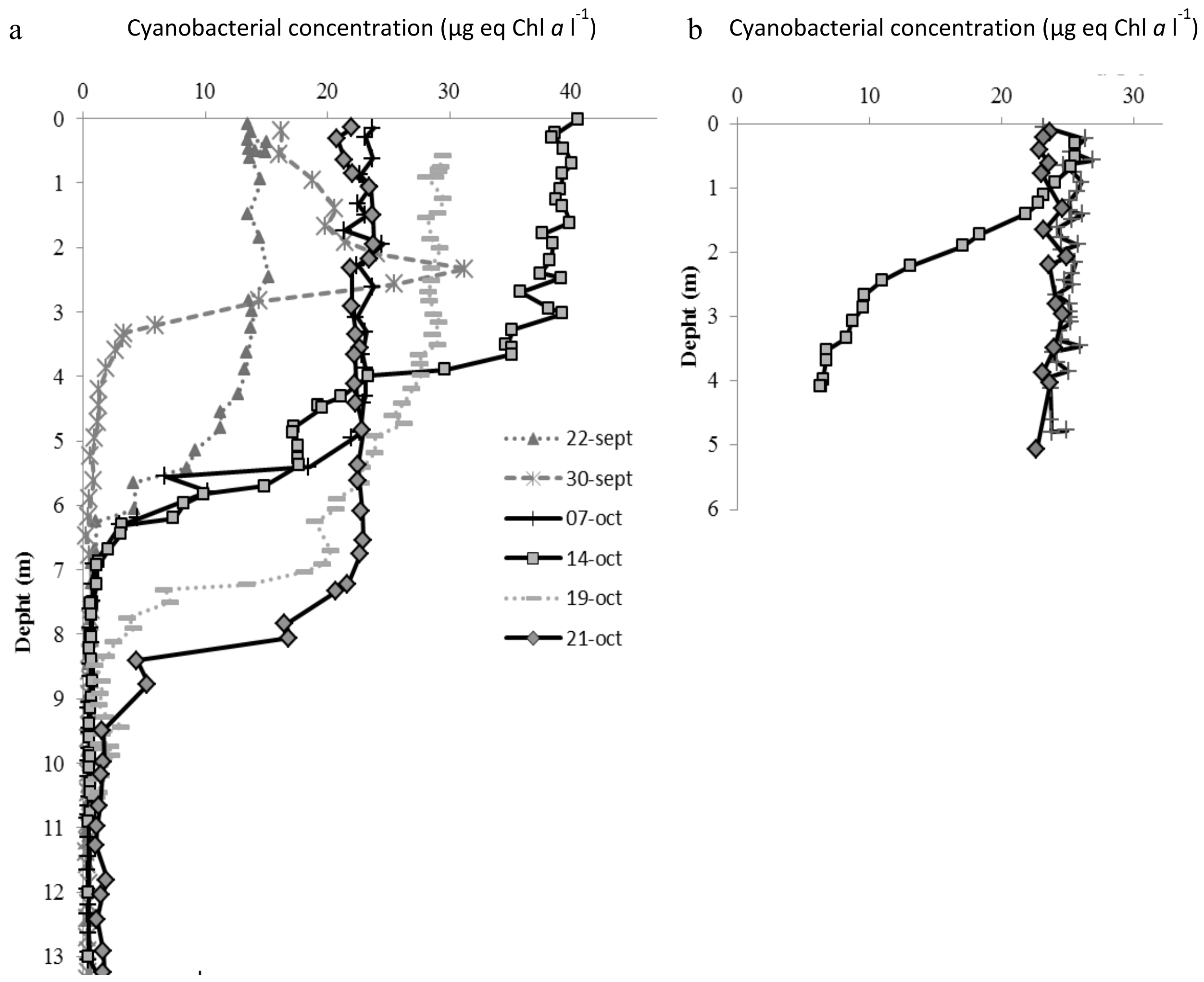

Supplementary Figure 3: Chlorophyll concentration profiles (from cyanobacteria) in October 2011 from the center (A) and the littoral (B) station of lake Aydat. Black lines represent sampled dates, others are intermediate dates.
